# Molecular Detection and Genetic Characterization of Two Dugbe Orthonairovirus Isolates Detected from Ticks in Southern Senegal

**DOI:** 10.3390/v16060964

**Published:** 2024-06-15

**Authors:** Mignane Ndiaye, Aminata Badji, Idrissa Dieng, Anna S. Dolgova, Moufid Mhamadi, Anastasiia D. Kirichenko, Anna S. Gladkikh, Alioune Gaye, Ousmane Faye, Amadou Alpha Sall, Mawlouth Diallo, Vladimir G. Dedkov, Oumar Faye

**Affiliations:** 1 Institut Pasteur de Dakar, 36 Avenue Pasteur, BP 220, Dakar 12000, Senegal; 2 St. Petersburg Pasteur Institute, Federal Service for Consumer Rights Protection and Human Well-Being Surveillance, 197101 St. Petersburg, Russia; 3 Martsinovsky Institute of Medical Parasitology, Tropical and Vector Borne Diseases, Sechenov First Moscow State Medical University, 119991 Moscow, Russia

**Keywords:** Dugbe orthonairovirus, ticks, phylogenetic analysis, Senegal, sequencing

## Abstract

Dugbe virus (DUGV) is a tick-borne arbovirus first isolated in Nigeria in 1964. It has been detected in many African countries using such diverse methods as serological tests, virus isolation, and molecular detection. In Senegal, reports of DUGV isolates mainly occurred in the 1970s and 1980s. Here, we report a contemporary detection of three novel DUGV isolates upon screening of a total of 2877 individual ticks regrouped into 844 pools. The three positive pools were identified as *Amblyomma variegatum*, the main known vector of DUGV, collected in the southern part of the country (Kolda region). Interestingly, phylogenetic analysis indicates that the newly sequenced isolates are globally related to the previously characterized isolates in West Africa, thus highlighting potentially endemic, unnoticed viral transmission. This study was also an opportunity to develop a rapid and affordable protocol for full-genome sequencing of DUGV using nanopore technology. The results suggest a relatively low mutation rate and relatively conservative evolution of DUGV isolates.

## 1. Introduction

Arboviruses are a group of viruses transmitted by arthropods [1]. They depend on vertebrate hosts as well as arthropod vectors such as ticks, mosquitoes, sandflies, and midges for their proliferation [2,3]. After mosquitoes, ticks are considered to be one of the main vectors transmitting arboviruses and other pathogenetic agents, such as bacterial and parasitic pathogens, that can cause severe illnesses to animals and humans [4,5]. Their role in the maintenance and dissemination of some of these viruses is considerable, acting both as vectors and reservoirs [6]. Therefore, tick-borne diseases (TBDs) represent a significant threat to human and animal health with global distribution [5].

It is proved that representatives of the *Orthonairovirus* genus are mainly transmitted by ticks [7]. However, no known vectors have been described for orthonairoviruses belonging to the Thiafora genetic group [8]. The genus *Orthonairovirus* includes viruses such as Crimean-Congo hemorrhagic fever virus (CCHFV), Nairobi sheep disease virus, Dugbe virus (DUGV), Hazara virus, and Kupe virus [9]. DUGV was first isolated in Nigeria in 1964 from a tick pool of *Amblyomma variegatum*, which is likely the main vector of DUGV [10,11,12]. DUGV is likely maintained in the environment by many other tick species (*Hyalomma* and *Rhipicephalus* species), as well as by livestock (cattle, goats, sheep, camels) and other vertebrates such as monkeys and birds. Humans are known to be susceptible to DUGV infection, displaying a mild febrile illness [13,14,15]. DUGV features a single-stranded, negative-sense RNA genome in three segments [16]. These three segments are designated as small (S), medium (M), and large (L). Their lengths are 1716 nucleotides (nt), 4888 nt, and 12,255 nt, respectively, with each containing a single open reading frame [16,17].

The geographical distribution of DUGV covers many countries in Africa, including Cameroon, Central African Republic, Chad, Egypt, Ethiopia, Ghana, Guinea, Kenya, Nigeria, Senegal, South Africa, Sudan, and Uganda [15,17,18,19,20,21]. In Senegal, the presence of DUGV has not been reported since the 1970s, when the majority of studies were carried out. Since then, no studies of DUGV distribution have been carried out in the country (https://wwwn.cdc.gov/Arbocat/VirusDetails.aspx?ID=135&SID=14, accessed on 14 December 2022). However, no human cases have been documented, despite the presence of potentially susceptible hosts and vectors. This is likely due to the absence of a surveillance system. In South Africa, one patient suffering from prolonged fever, headache, malaise, thrombocytopenia, orchitis, and signs of hemorrhage has been reported. Actual DUGV infection was confirmed by the detection of specific IgG antibodies [15]; this suggests a necessity to investigate DUGV in countries where it is present, such as Senegal.

In this study, we report the detection of DUGV in 2021 from ticks, despite decades without active surveillance in Senegal. The full sequence of the virus was obtained by sequencing the amplified genome with a multiplex tilling RT-PCR approach, followed by library preparation based on Nanopore technology. Phylogenetic analysis was carried out on the obtained sequences in order to explore the evolutionary relationships between newly and previously identified DUGV isolates.

## 2. Materials and Methods

### 2.1. Study Area

**Figure 1:**
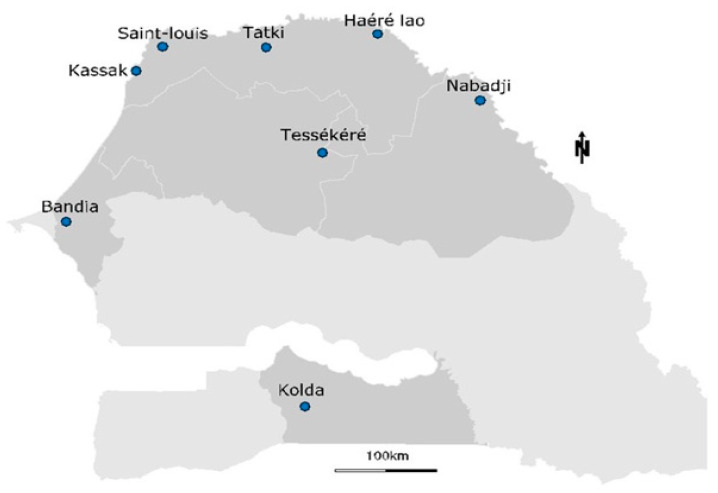
Map showing tick sampling locations around Senegal. Ticks were collected from eight localities belonging to five administrative regions.

The study area included various geographical regions of the country, including a semi-desert zone with an annual rainfall of around 300 mm in the north and a forest zone characterized by an annual rainfall of up to 1000 mm in the south (Figure 1). The other sampling sites were located in the center of the country featuring a wooded savannah. In the northern and central regions, temperatures vary from 20 °C on the coast to 48 °C inland, depending on the season. The southern regions record minimum temperatures of around 22 °C and maximum temperatures of 38 °C, mainly during the dry season. Overall, eight sampling sites were arbitrarily selected, and sampling was performed in July 2021 during the rainy season.

### 2.2. Tick Collection and Identification

We visited 24 herds with a total of 720 animals, including 240 cattle, 240 sheep, and 240 goats kept under semi-extensive conditions. The animals were kept in cattle sheds and sheepfolds at night. During the day, they were turned out onto pastures around the villages with access to a watering point for drinking. An average of 30 animals were randomly selected from each farm during each visit. Ticks found on the skin surfaces of diverse animals were collected in clean containers and immediately put in dry ice for transport to the Laboratory of Medical Zoology (Institute Pasteur in Dakar, Dakar, Senegal) for analysis. At the laboratory, ticks were initially washed using phosphate-buffered saline and identified based on morphological characteristics following the criteria of Walker et al. (2014) [22]. The identified ticks were grouped into monospecific pools of 1 to 28 individuals according to location, sex, date, and feeding status. All samples were then frozen in otherwise empty tubes and stored (−80 °C) until further analysis.

### 2.3. Tick Homogenization, RNA Extraction, and RT-PCR

Identified ticks were homogenized individually with Leibovitz-15 Medium using a TissueLyser II (Qiagen, Hilden, Germany) and metallic beads, followed by regrouping into pools. The homogenate was then centrifuged at 10,000 rpm for 10 min (+4 °C). The supernatant was harvested into cryotubes and stored at −80 °C before analysis. RNA extraction was performed using 140 µL of supernatant with the QIAamp Viral RNA Mini Kit (Qiagen, Hilden, Germany) according to manufacturer recommendations. To detect whether DUGV was present in tick pools, an RT-PCR reaction was performed using the AgPath-ID One-step RT-PCR kit (ThermoFisher Scientific, Waltham, MA, USA) in a CFX96 Real-Time PCR Detection System (Bio-Rad Laboratories, Hercules, CA, USA). The primer/probe system (based on the S segment) used for the detection of DUGV in ticks has been described previously [21].

### 2.4. Nanopore Sequencing

The reverse transcription reaction was performed using random hexanucleotide primers and a Reverta-L kit (AmpliSens, Moscou, Russia) according to the manufacturer’s instructions. The cDNA was stored at −70 °C and used subsequently as a template for amplification. Due to the low Ct value of the third positive pool, only the three segments of the DUGV genome present in the two positive tick pools with low Ct values (Ct < 28) were successfully amplified using a web-based primer design tool—Primal Scheme (http://primal.zibraproject.org, accessed on 14 April 2022). This tool offers a complete pipeline for the development of efficient multiplex primer schemes for the generation of overlapping products, the size of which is determined by the target genome length, amplicon length, and required overlap. Analogous approaches have been described earlier for the Dengue virus (DENV) [23], Zika [24], Paramushir virus [25], and SARS-CoV-2 [26]. PCR reactions were performed as previously described [27]. Amplicons generated were then purified at a 1:1 ratio with Ampure beads (Beckman Coulter, Brea, CA, USA). Libraries were prepared according to a rapid sequencing DNA barcoding (SQK-RBK110.96) protocol (Oxford Nanopore, Oxford, UK). Briefly, purified amplicons were tagged with Rapid Barcodes (RB01-96) incorporated into the Rapid Barcoding Kit 96 (above). Sequencing was performed using an MK1C sequencer (Oxford Nanopore MinION) by loading the pools of tagged samples into R 9.4.1 flow cells (FLO-MIN111). The sequencing reaction was performed for at least 24 h.

### 2.5. In Silico Analysis

After sequencing, raw data were base-called using Guppy version 3.2.10 (Oxford Nanopore Technologies); analysis was performed with an in-house pipeline. Briefly, fastq files were trimmed using Nanofilt v.2.8.0 (Option –headcrop 50, –tailcrop 50) [28]. The obtained trimmed reads were then mapped to corresponding Dugbe segment reference sequences (MW917186-MW917188) using Minimap v.2.24 [29]. Sequence alignment map (SAM) files were sorted and transformed into a binary alignment map (BAM) file using SAMtools v.1.18. BAM files were manually visualized using Tablet v.1.21.02.08 [30] to identify potential erroneous variants. Consensus sequences were finally generated in BEDtools v.2.28.0 [31] by masking regions with a depth lower than 20×.

Genome annotation of DUGV isolates was performed using The Genome Annotation Service and the Viral Genome ORF Reader (VIGOR v.4.0) (https://github.com/JCVenterInstitute/VIGOR4/releases, accessed on 13 January 2023). DUGV isolate sequences newly obtained and previously published in GenBank were aligned and analyzed using MEGA v. 11 software [32] with the muscle algorithm for multiple alignments, GBlocks v.0.91b [33] to close gaps in multiple alignments, and jModelTest v.2.1.10 to select the optimal nucleotide substitution model for each segment [34,35]. Phylogenetic trees were constructed using the PhyML method (v3.1) with Seaview (v5.0.5) [36]. The robustness of trees was tested using 1000 bootstrap replicates. Trees were drawn in Figtree v1.4.4 (http://tree.bio.ed.ac.uk/software/figtree/, accessed on 13 January 2023).

A test for probable recombination events was performed with SimPlot v3.5.1.0 [37] software and with the Recombination Detection Program (RDP) v4.101 [38] using eight methods provided by the software. The availability and location of signal peptides in translated nucleotide sequences were predicted using SignalP v5.0 software (https://services.healthtech.dtu.dk/services/SignalP-5.0/, accessed on 16 January 2023) [39]. Glycoprotein secondary structures were predicted using PSIPRED v3.3 software [40]. All molecular graphics were produced using PyMOL software (PyMOL Molecular Graphics System v1.8) (https://github.com/schrodinger/pymol-open-source, accessed on 17 January 2023).

## 3. Results

### 3.1. Tick Collection

**Figure A1:**
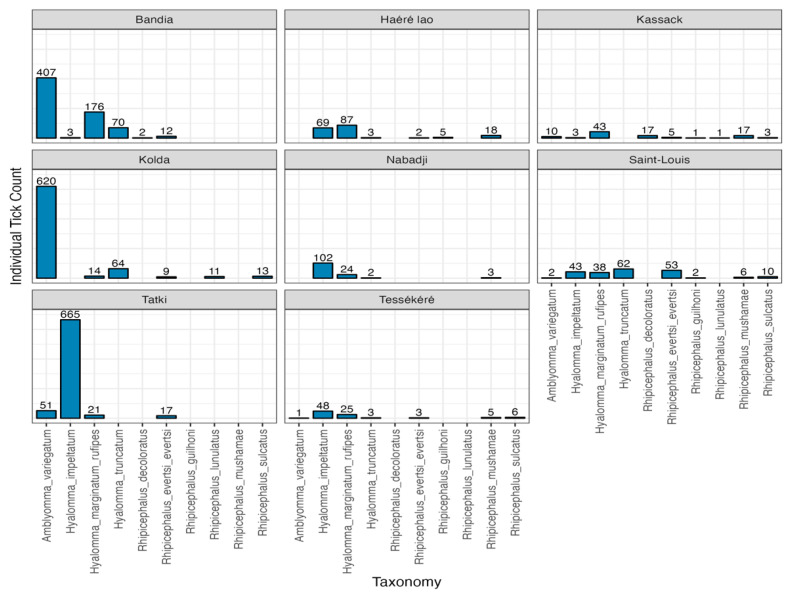
Spatial and taxonomic distribution of sampled ticks, including the number of individual ticks.

**Figure A2:**
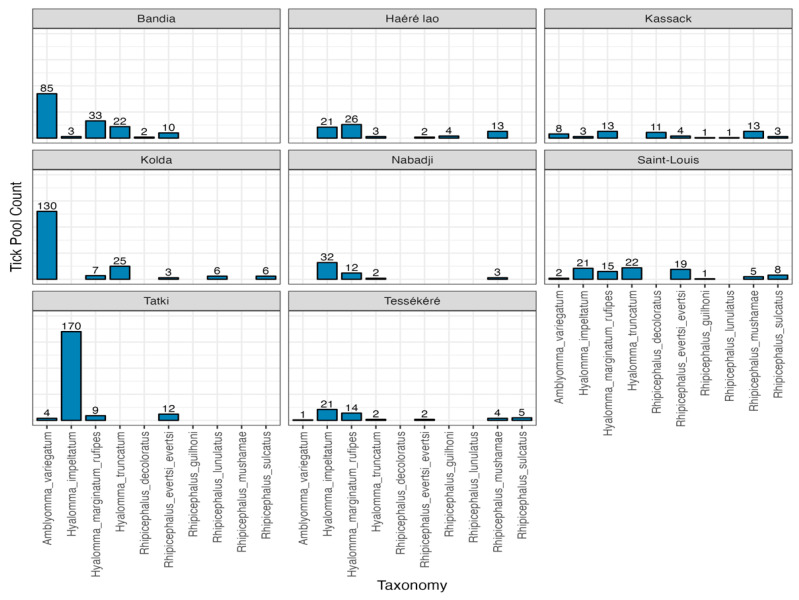
Spatial and taxonomic distribution of sampled ticks, including the number of tick pools.

A total of 2877 ticks were collected (adults, nymphs, or larvae) in the different areas, representing ten species (*A. variegatum*, *Hyalomma impeltatum*, *H. rufipes*, *H. truncatum*, *Rhipicephalus decoloratus*, *R. evertsi evertsi*, *R. guilhoni*, *R. lunulatus*, *R. mushamae*, *R. sulcatus*). Tick species of the genus Hyalomma were predominant (54.4%), followed by the genera *Amblyomma* (37.9%) and *Rhipicephalus* (7.7%). *Hyalomma* and *Rhipicephalus* ticks were present at all eight sampling sites, while *Amblyomma* ticks were present at six of the eight sites (Figure A1). The number of pools obtained and tested after identification was equal to 844 pools based on species and the host of collection (Figure A2).

### 3.2. DUGV Detection in Ticks

According to RT-PCR results obtained by screening with the primer/probe system described previously [21], the presence of DUGV was confirmed in 3 pools out of a total of 844 pools tested. These positive pools consisted of six, three, and one individuals identified as the tick species *Amblyomma variegatum*. Geographically, these positive pools were all collected in the Kolda region in the south of the country at the border with Guinea Conakry and Guinea-Bissau. DUGV was not detected in any other tick species screened (Table 1).

### 3.3. Genetic Analysis

For two out of the three DUGV-positive pools analyzed (IDs 2035, 2125) with low Ct values (Ct < 28), near-complete genomes were successfully sequenced. These sequences were designated as Dugbe orthonairovirus isolate 2035_RSF and Dugbe orthonairovirus isolate 2125_RSF (abbreviated as Dugbe_2035, Dugbe_2125). For isolates Dugbe_2035 and Dugbe_2125, 145,379 and 158,665 reads were achieved in total after trimming, respectively. After processing through the pipeline, reads were mapped to segments as follows for isolate Dugbe_2035: 31,713 to L, 41,010 to M, and 29,524 to S. For isolate Dugbe_2125, mapping was as follows: 37,466 to L, 32,321 to M, and 33,651 to S. For Dugbe_2035, genomic coverage with sequencing depth more than 20 was 93.1% for the L segment, 99.8% for M, and 100% for S. For Dugbe_2125, this parameter was 82.2% for the L segment, 99.1% for M, and 100% for S. Sequences were submitted to GenBank under accession numbers OQ557148-OQ557153.

The DUGV_2035 and DUGV_2125 isolates differ from each other in the S segment at eight nucleotide positions; one leads to a nonsynonymous substitution. In the M segment, 12 positions differ (4 nonsynonymous). In the L segment, 33 positions differ (1 nonsynonymous). In addition, DUGV_2035 has eight unique single nucleotide variations (SNVs) in the L segment relative to other known isolates. The M segment features four unique SNVs. There are two unique SNVs in the S segment. DUGV_2125 features seven unique SNVs in the L segment (2 nonsynonymous), no unique SNVs in the M segment, and one unique (nonsynonymous) SNV in the S segment (Table 2).

**Figure 2:**
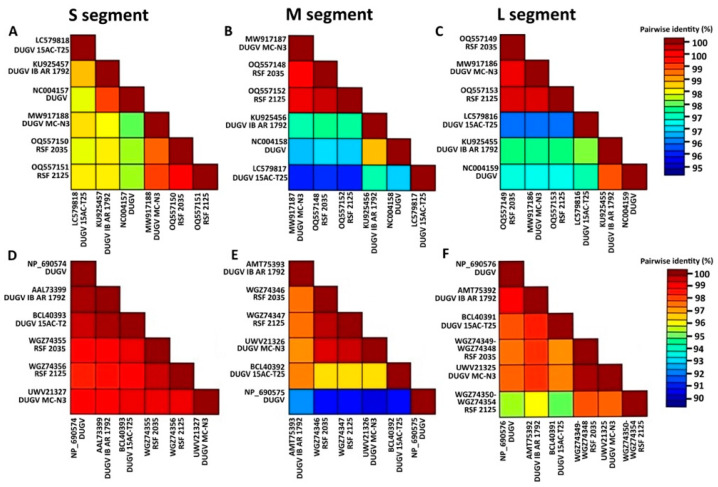
Sequence identity heatmap showing pairwise percent identity in nucleotide and amino acid composition between S segments (**A**,**D**), M segments (**B**,**E**), and L segments (**C**,**F**) of all known DUGV isolates, respectively. Heatmaps were drawn using SDT v 1.2. software [42]. Based on the heatmap, over 95% nucleic acid identity in all segments can be assumed. At the amino acid level, S segment nucleocapsid proteins of all strains are more than 98% similar. The M segment polyprotein is more than 95% similar in all isolates, with the exception of DUGV isolate (NP_690575), which is 90–92% similar to the others. The similarity of the RNA-depended RNA polymerase, encoded in the L segment, is 97% or higher for all isolates, with the exception of isolate RSF_2125, which has about 97% identity with isolates DUGV_2035 and MC-N3. Two novel isolates (DUGV_2035, DUGV_2125) and DUGV isolate MC-N3, obtained in Ghana from *A. variegatum* ticks, shared more than 99% amino acid identity in each segment.

Currently known DUGV isolates are extremely close, sharing over 95% nucleic acid identity in all segments. Based on amino acid composition, it can be noted that the S segment nucleocapsid protein of all isolates is more than 98% similar. The M segment polyprotein is more than 95% similar in all isolates, with the exception of a DUGV isolate (NP_690575), which is 90–92% similar to the others. The similarity of the RNA-depended RNA polymerase (encoded in the L segment) is 97% or higher for all isolates, with the exception of our isolate RSF_2125, which also has high similarity with isolates DUGV_2035 and MC-N3 (about 97% identity); the similarity with other isolates is slightly lower (94.5–96%). In addition, the two novel isolates (DUGV_2035, DUGV_2125) and DUGV isolate MC-N3 (acc. Nos. MW917186-MW917188) shared more than 99% identity in each segment (Figure 2). MC-N3 was previously obtained in Ghana from *A. variegatum* ticks.

**Figure 3:**
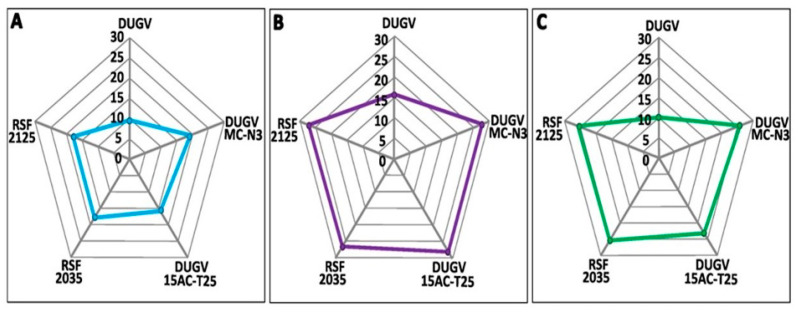
Radar plots representing the number of substitutions that have occurred in the small ((**A**), blue), medium ((**B**), purple), and large ((**C**), green) genomic segments. The sequence of isolate IB AR 1792 was used as a reference, retrieved from the oldest collection (dated 1964). The number of nucleotide substitutions in the M segment of DUGV strains is greater than that in the L or S segments. Isolates DUGV_2035, DUGV_2125, and MC-N3 could be considered to constitute the most genetically divergent group in comparison with DUGV isolate IB AR 1792.

Based on radar plot analysis, the number of nucleotide substitutions in the M segment of DUGVs is greater than that in the L or S segments. Thus, the M segment was found to be the most genetically divergent. Moreover, isolates DUGV_2035, DUGV_2125, and MC-N3 could be considered to constitute the most genetically divergent group in comparison with DUGV isolate IB AR 1792 (acc. Nos. KU925455-KU925457). Isolate IB AR 1792 was obtained in Nigeria from *A. variegatum* ticks, retrieved from the oldest collection, dated 1964 (Figure 3). This assumption is supported by the phylogenetic analysis below.

**Figure 4:**
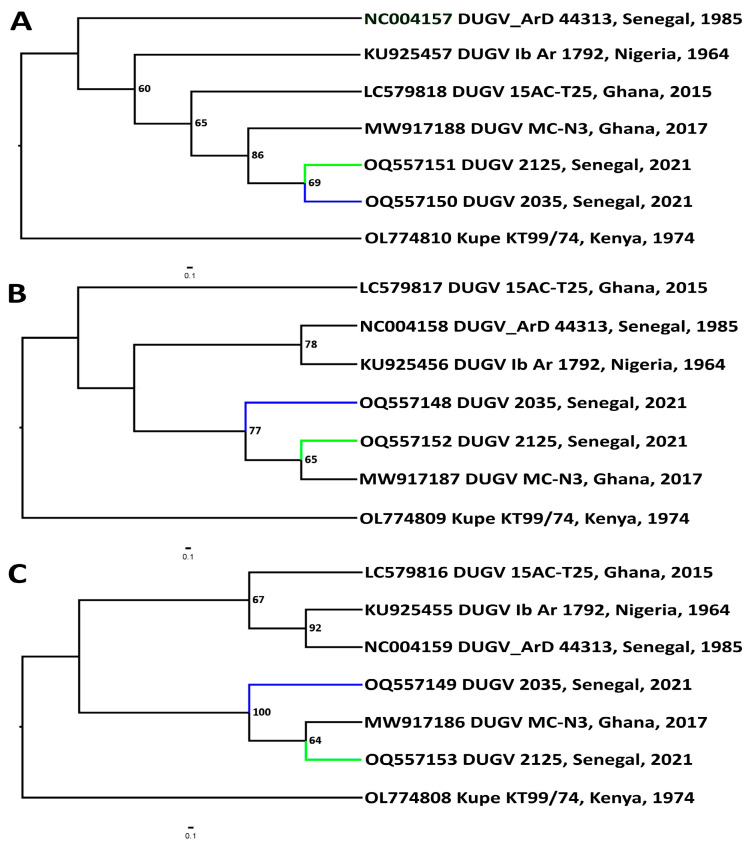
Dendrograms for near-complete Dugbe orthonairovirus genomic segments (nucleotide sequences). Phylogenetic analysis was performed using individual substitution models: for S segment, HKU + I; for M segment, GTR + I + G; and for L segment, GTR + G. The robustness of trees was tested using 1000 bootstrap replicates and numbers at the tree nodes represent bootstrap support values. Nucleotide sequences used in phylogenetic analysis were as follows (GenBank numbers in brackets): DUGV isolate ArD 44313 isolated in Senegal in 1985 (NC_004157-NC_004159), DUGV isolate Ib Ar 1792 isolated in Nigeria in 1964 (KU925455-KU925457), DUGV 15AC-T25 isolated in Ghana in 2015 (LC579816-LC579818), and DUGV isolate MC-N3 also isolated in Ghana in 2017 (MW917186-MW917188). The closely related Kupe virus isolate KT99/74, isolated in Kenya in 1974 (OL774808-OL774810), was used as an outgroup. (**A**) S segment sequences; (**B**) M segment sequences; (**C**) L segment sequences. The branches for isolates RSF_2035 (blue) and RSF_2125 (green) are highlighted.

Dendrograms of different genomic segments revealed distinct phylogenetic relationships between DUGV isolate RSF_2035, isolate RSF_2125, and isolate MC-N3 (Figure 4). Regarding the M and L segments, isolate RSF_2125 and isolate MC-N3 form a well-supported clade related to isolate RSF_2035. The reference DUGV isolate forms a reliably supported clade with isolate Ib Ar 1792 in the L and M segments. In the L segment, they are also related to isolate 15AC-T25. For the M segment, isolate 15AC-T25 is an outgroup for all other DUGV isolates analyzed phylogenetically. For the S segment, all DUGV isolates were sequentially grouped into a single clade. This suggests a common origin, with isolates RSF_2035 and RSF_2125 in this case being closest neighbors and moving away from the terminal node.

Bootscan analysis using Simplot software v3.5.1 also showed that Dugbe viruses are very close, with a similarity of over 95% in all segments. There were no probable recombination events. An analysis performed by RDP v4.101 confirmed an absence of probable recombination events in all segments. Probable recombination events were not found in any of the sequenced strains.

**Figure 5:**
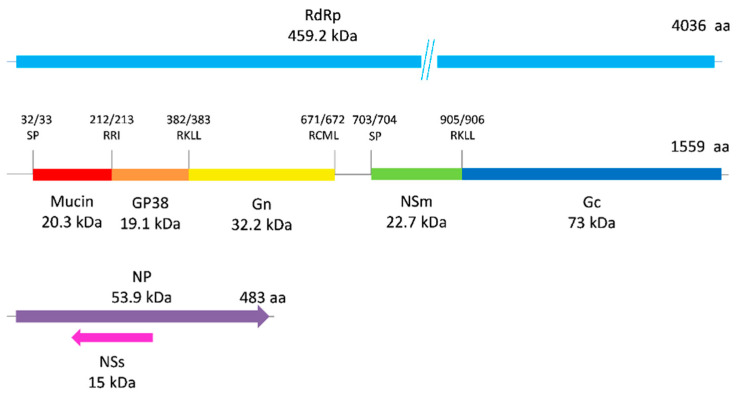
Proteomic organization. Predicted signal peptidase (SP) cleavage sites and putative cleavage sites of the M segment are labeled. Regions corresponding to the RdRp (light blue), Mucin (red), GP38 (orange), Gn (yellow), NSm (green), Gc (blue), NP (purple) and NSs (pink) are shaded. Reading frames of NP and NSs are shown by arrows.

The obtained full genomic sequences enabled the prediction of proteomic organization for the Dugbe isolates. DUGV L segment amino acid sequences allow us to assume that RdRp is 4036 aa in length and about 459.2 kDa in size. The M segment encodes a 1559 aa glycoprotein precursor 174 kDa in size. The S segment encodes nucleoprotein (NP), 483 aa in length and 53.9 kDa in size (Figure 5).

**Figure 6:**
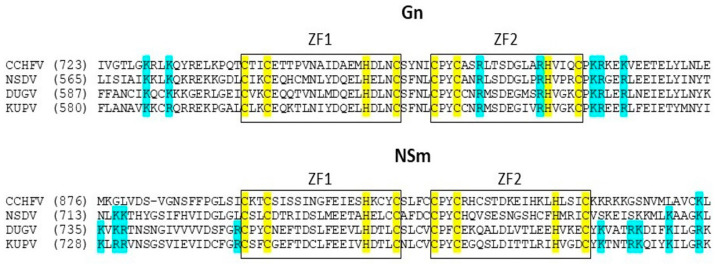
Sequence alignment of the Gn tails and N-termini of NSm for DUGV and *Bunyaviridae* family members. Conserved zinc finger motifs are boxed. The strictly conserved cysteine and histidine residues and the group of conserved basic residues are highlighted in yellow and blue respectively.

The Gn tail of the Dugbe virus contains ββα-type zinc finger motifs. Conserved CXCXHXC motifs of cysteine and histidine (where X is any amino acid), characteristic of ββα-type zinc fingers, are also present at the N-terminus of the NSm protein. It can be seen that CCHFV also has such motifs at this site, but there are no characteristic basic residues in the vicinity of the CCHC motifs. They are also present in DUGV and Kupe virus (KUPV) (Figure 6).

**Figure 7:**
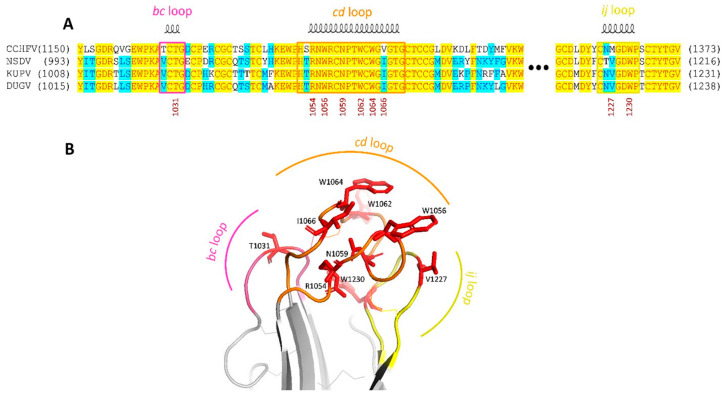
Comparative fusion loop sequence and structure. (**A**) Structure-based multiple sequence alignment of fusion loop regions. Strictly conserved and identical residues are highlighted in yellow and blue, respectively. The residues correspond to polyprotein precursor numbering. (**B**) Putative Dugbe virus fusion loops. Hydrophobic residues that anchor the protein to the cellular membrane are shown in red, with disulfide bonds in stick representation.

A close amino acid composition of the fusion loop structure is found between the CCHF virus, DUGV, and their closest relatives (NSDV, KUPV) (Figure 7A). Indeed, based on the alignment and spatial structure (Figure 7A,B), the basic amino acids are highly conserved.

## 4. Discussion

DUGV is an arbovirus transmitted by ticks, mainly *Amblyomma variegatum*, which belongs to the genus *Orthonairovirus,* family *Nairoviridae* [43]. Due to its epidemic potential [21,44], we investigated the prevalence of DUGV in vectors, namely ticks, in this study. We detected three isolates of DUGV and highlighted the presence of this infectious agent after around thirty years without available information. All three isolates of DUGV were found in pools of ticks of the species *Amblyomma variegatum*, which is known to be the main vector of DUGV with possible transstadial and transovarian transmission. Thus, this tick species seems to be the main reservoir of DUGV and therefore the species of ticks responsible for maintaining it in nature in Senegal.

Another striking fact is the significant proportion occupied by ticks of the genus *Hyalomma* among our samples, which has already been confirmed in Senegal by previous studies [45,46]. This type of tick is known to be the vector and reservoir of CCHFV, an orthonairovirus that can cause significant health harm in humans [47]. Thus, given the emergence of arboviruses in recent years, surveillance of these pathogenic agents could make it possible to determine the zones at risk in order to avoid possible epidemic outbreaks.

Phylogenetic trees were constructed by the maximum-likelihood method based on the genome sequences (S, M, L segments) obtained in this study and other sequences downloaded from GenBank. Phylogenetic analysis first showed that the two newly isolated isolates were very similar. Subsequently, by comparing them with isolates isolated in Senegal and in other countries, we found great similarity between the reference isolate isolated in Nigeria and the three isolates that have been isolated in Senegal [17]. On the other hand, L segment analysis showed greater similarity to isolates from Kenya. Therefore, these results overall suggest a relatively low mutation rate and relatively conservative evolution in DUGV compared to other RNA viruses like CCHFV [48]. Based on radar plot analysis, isolates DUGV_2035, DUGV_2125, and MC-N3 could be considered to constitute the most genetically divergent group in comparison with DUGV isolate IB AR 1792. These three isolates are among the most recently reported strains of DUGV. In CCHFV, strains isolated from similar regions decades apart show strong sequence conservation suggesting a limited temporal evolution of CCHFV within geographical regions [49,50]. However, further investigations are needed to understand the impact of temporal pressure on the genetic evolution of DUGV.

The L segment of DUGV, encoding an RNA-dependent RNA polymerase (RdRp), is highly conserved. The M segment proteins of the DUGV_2035 and DUGV_2125 isolates are very similar. The differences are only four aa for all precursors. It is important to note that in the reference isolate (NC_004158_DUGV ArD44313), the genomic M segment is most likely not assembled correctly. There are differences from all other genomes in significant areas, such as in the fusion loop region. Since the article describing the isolate is from 1992 [16], it can be assumed that there could be assembly errors.

As mentioned above, the M segment encodes a glycoprotein precursor. For some representatives of *Nairoviridae*, it is believed that this precursor is subsequently cleaved into three parts: G1, Gn, and Gc [16,41]. However, for the Crimean-Congo hemorrhagic fever virus (CCHFV), it is generally accepted that G1 is also separated into mucin and GP38 proteins and that the NSm protein is cleaved from the Gn protein C-terminus [51,52,53].

It is likely that the situation is the same for DUGV. CCHFV mucin/GP38 cleavage is performed with the involvement of the RSKR furin cleavage site [53]. The Eukaryotic Linear Motif (ELM) resource [54] found the N-Arg dibasic convertase (nardilysin) cleavage site 210RRI212 in the same location in Dugbe virus. The ELM resource describes this site as an NRD convertase recognition motif in *Rattus norvegicus*. It is probable that human nardilysin is analogous to NRD convertase, with the ability to cleave peptides containing an arginine dibasic pair [55]. This could indicate the possibility of enzymatic formation of mucin and GP38 in DUGV. Moreover, even if this does not happen, it has been shown that CCHFV mutants lacking this furin site (thereby lacking optimal GP38 release) have only slightly decreased Gn maturation as well as a transient reduction in viral titers. This is a sign that the furin site is not strictly required for viral replication [53].

In CCHFV, after NSm is cut off from the C-terminus of Gn, a signal peptide is released, along which the final formation of NSm occurs. Overall, SignalIP with 0.6567 likelihood shows a signal peptide (Sec/SPI) recognition site with a cut after 703 Gly in DUGV. The position of the site corresponds to that in CCHFV NSm. Unlike CCHFV, the SKI-I cleavage site 804RKLL807 in DUGV is replaced with 668RCML671. This site, however, is also possible for SKI-I cleavage at the conserved [RK].[AILMFV][LTKF] motif at the 671/672 amino acid position. In hamster SREBP protein, for example, Met also occupies the third position. According to certain studies, the second and third positions are generally of little importance and could be replaced by Ala without loss of activity [56]. As such, it is possible that cutting also occurs here, and the NSm protein is obtained, as has been suggested [57].

In general, it is highly probable that the maturation of protein molecules in DUGV occurs in the same way as that in CCHFV. Considering the results of El Ghorr and colleagues (1990) [58], we can draw the following conclusions about DUGV structural proteins: The Dugbe virus was grown in a pig kidney epithelial (PS) cell line. Western blotting and probing with a rabbit polyclonal antiserum to DUGV permitted the detection of seven different proteins, and one was about 200 kDa in size (174 kDa glycoprotein precursor). This fact supports our assumption of proteomic organization shown in Figure 5.

Based on the sequence alignment of the Gn tails (Figure 6), the presence of ββα-type zinc finger motifs in the DUGV Gn tail may suggest its involvement in interactions with viral RNA, as previously reported [59]. Upon further study, it turned out that conserved CXCXHXC motifs of cysteine and histidine (where X is any amino acid), which are characteristic of ββα-type zinc fingers, are also present at the N-terminus of the NSm protein. CCHFV also has such motifs at this location, but there are no characteristic basic residues in the vicinity of the CCHC motifs. They are present in DUGV and Kupe virus (KUPV). Zinc finger domains have already been found in non-structural proteins such as those from coronaviruses. Their functionality may be somehow related to RNA binding [60,61]. Perhaps in this case they could have the same function.

The Gc proteins of the two isolates differ by one aa (1488Phe/Leu). Based on the close amino acid composition to the most well-studied structure of the fusion loop of the CCHF virus (Figure 7A), it can be said that the DUGV and its most closely related relatives (NSDV, KUPV) presumably have the same mechanism of cell penetration. The basic amino acids are highly conserved; this can be seen in alignment and spatial structure (Figure 7A,B). A 3D model was built on the X-ray structure of the CCHFV Gc ectodomain in the post-fusion conformation, SMTL ID: 7a5a.1 [62]. It can be said from it that DUGV Gc could have a similar tripartite configuration with typical class II fold loops (bc, cd, ij) which form a nonpolar host–membrane insertion surface (HMIS) required to drive membrane fusion. Of the processes involved in fusion, the differences are in 1066Ile (1201Val in CCHFV) and 1227Val (1362 Met in CCHFV). Only experimental tests could tell how significant these replacements are.

In the S segment, in addition to NP, there may also be an NSs protein in the opposite reading frame (139 aa, 15 kDa). Barnwal and colleagues (2016) [63] showed that CCHFV has such a protein; it is responsible for disrupting mitochondrial membrane potential; and it induces apoptosis. DUGV NSs has 38% identical and 46% consensus aa with it. Important for the described activity were aa Leu-127 and Leu-135. The consensus Leu-135 in DUGV is represented by Leu-137, but instead of Leu-127, there is Cys-129. Whether it carries any activity is not yet clear. There is another reading frame on the positive sense of the S segment genome. It could be translated into a 9 kDa protein with 84 aa, but it is not yet clear whether it actually exists. However, if we return to the 1990 publication again [58], there is a small band that the authors do not consider a possible protein. There is a chance that it could be just this protein. However, it should be noted that our assumptions about DUGV protein structures and their properties require further verification.

In Senegal, the majority of DUGV findings were carried out in the center of the country in the Bandia area (catalogue of isolated arboviruses at Institut Pasteur de Dakar (offline)). Curiously, every isolate of DUGV was not detected in this area in our study, which could suggest a regressive evolution of this virus in this locality. However, information about the state of animal transhumance is unknown. This constitutes a limitation of our study insofar as we cannot determine the exact origin of the isolates. Nevertheless, we report the first DUGV detection in this southern part of the country to our knowledge. Consequently, global climate change could perhaps play an important role in the dynamics of virus circulation.

Recently, a case of DENV was reported in this southern area [64], along with CCHF (unpublished data). The region was considered to be an area with a low prevalence or even a complete lack of arboviruses. It would be therefore interesting to strengthen surveillance in this part of the country. No human cases of DUGV have been reported since it was first confirmed in the country in 1973 [17]. Our report suggests that DUGV has persisted in nature for more than four decades. Thus, since humans are known to be susceptible to DUGV infection, active surveillance for this orthonairovirus may serve to fill this data gap while assisting public health efforts.

The multiplex primer sets used in this study appear to be efficient for DUGV sequencing using the Oxford Nanopore Technologies platform. In this regard, knowing the many advantages offered by this technology in terms of economy, efficiency, and portability, it would be interesting to test them on a larger number of isolates covering a greater diversity.

## Figures and Tables

**Table 1:** Molecular detection of DUGV by tick species and sampling area. Northern areas included Nabadji, Haere Lao, Tatki, Tessekere, Kassack, and Saint-Louis.

Sampling Site	Tick Species	Number of Pools Tested	Numbers of Positive Pools (%)
Northern areas (Nabadji, Haere Lao, Tatki, Tessekere, Kassack, Saint-Louis)	*Hyalomma* spp.	386	0 (0%)
	*Amblyomma variegatum*	15	0 (0%)
	*Rhipicephalus* spp.	111	0 (0%)
Central area (Bandia)	*Hyalomma* spp.	58	0 (0%)
	*Amblyomma variegatum*	85	0 (0%)
	*Rhipicephalus* spp.	12	0 (0%)
Southern area (Kolda)	*Hyalomma* spp.	32	0 (0%)
	*Amblyomma variegatum*	130	3 (2.3%)
	*Rhipicephalus* spp.	15	0 (0%)

**Table 2:** Number of SNVs per segment of the different isolates compared to the DUGV reference isolate, IB AR 1792 (acc. numbers KU925455-KU925457), obtained in Ibadan, Oyo State, and Nigeria in 1964 [41].

	Isolate ArD 44313			Isolate 15AC-T25			Isolate MC-N3			Isolate 1			Isolate 2		
	S (NC_004157)	M (NC_004158)	L (NC_004159)	S (LC579816)	M (LC579817)	L (LC579818)	S (MW917186)	M (MW917187)	L (MW917188)	S (OQ557151)	M (OQ557152)	L (OQ557153)	S (OQ557148)	M (OQ557149)	L (OQ557150)
**SNVs (vs. REF)**	14	73	105	23	128	241	28	128	268	26	121	263	26	124	267
**nonsynonymous SNVs (vs. REF)**	3	44	75	1	79	175	5	67	197	5	63	197	7	63	199

## Data Availability

Sequencing data are available in GenBank NCBI under accession numbers OQ557148-OQ557153.
